# Crosstalk of parkin and Ret in dopaminergic neurons

**DOI:** 10.18632/oncotarget.4465

**Published:** 2015-06-13

**Authors:** Edgar R. Kramer

**Affiliations:** Development and Maintenance of the Nervous System, Center for Molecular Neurobiology, University Medical Center Hamburg-Eppendorf, Hamburg, Germany

Recent genetic and functional studies have revealed striking similarities in the protein networks and molecular mechanisms altered in cancer and Parkinson's disease, as illustrated here for the converging signaling pathways of parkin and Ret [1]. These similarities may guide our thinking about potential therapies for both diseases.

The proto-oncogene RET encodes a receptor tyrosine kinase that is the canonical glial cell line-derived neurotrophic factor (GDNF) family receptor. The name stands for “rearranged during transfection” since Ret was found to be fused to a putative zinc finger protein rfp *in vitro* during transfection of human T-cell lymphoma DNA in 3T3 fibroblasts [2]. In the meantime, oncogenic activating mutations in Ret were found to cause multiple endocrine neoplasia type 2 (MEN2), a dominant inherited cancer syndrome that affects neuroendocrine organs such as the thyroid. Conversely, loss-of-function mutations are the main cause of Hirschsprung disease, a congenital absence of enteric ganglia in the hindgut. Interestingly, some mutations can even lead to MEN2 and Hirschsprung disease simultaneously [3]. Ret/GDNF signaling has also been shown to have many important functions in the mammalian body, including affecting the survival of midbrain dopaminergic neurons of the substantia nigra, which preferentially die in Parkinson's disease (PD) patients [2]. Thus far, we could show in three different Ret-deficient mouse lines that specifically substantia nigra dopaminergic neurons die progressively with age, suggesting a cell-autonomous maintenance function of Ret in these neurons [4, 5]. To date no Ret mutations were found in PD patients, most likely because Ret mutations are frequently life-threatening. But recently we could show that Ret is tightly linked to the protein network altered in PD patients and crosstalks directly with proteins like the redox-dependent molecular chaperone and oncogene DJ-1 and the E3 ubiquitin protein ligase and tumor suppressor protein parkin [5, 6].

Parkin is encoded by the PARK2 gene and was originally identified as a gene mutated in some familial forms of PD. In the meantime, PARK2 mutations have also been found in sporadic forms of PD and account for most autosomal-recessive PD cases [7]. Parkin mutations were also shown to lead to glioblastoma, breast, colon, pancreas, liver and lung cancer [1]. Interestingly, the same mutations could be found in PD and cancer patients, also heterozygosity is sufficient to trigger uncontrolled cancerous cell division, while in PD patients both copies need to be mutated [1].

To investigate a potential crosstalk of parkin and Ret we generate two mouse models and analyzed their phenotype. The first mouse model was parkin and Ret double-deficient mice which showed an accelerated dopaminergic cell loss specifically in the substantia nigra and dopaminergic fiber loss in the striatum compared to no alterations in parkin- and a moderate degeneration in Ret-deficient mice [5]. The double deficient mice also showed reduced dopamine levels, increased anxiety and reduced ATP and mitochondrial complex I activity as also reported for PD patients. These mice revealed for parkin a dopaminergic cell survival and axon maintenance function in the absence of the neurotrophic receptor Ret. The second mouse model was mice overexpressing human parkin in a Ret-deficient background which did not show the age-related dopaminergic system degeneration observed in Ret-deficient mice. This suggests that enhanced parkin signaling is not only protective against toxin- and stress-induced dopaminergic system degeneration, but also against neurotrophic deprivation induced by Ret loss.

To address the molecular mechanisms of this genetic crosstalk between parkin and Ret, we went ahead and focused on analyzing the effect on mitochondria in more detail [5]. We found that down-regulation of both parkin and Ret led to impaired mitochondrial function and morphology and that parkin and GDNF/Ret can substitute for each other to ensure proper mitochondrial function by converging signaling cascades activating the nuclear factor ‘kappa-light-chain-enhancer’ of activated B-cells (NF-κB). Ret activates NF-κB through the phosphoinositid-3-kinase (PI3K) pathway. Taken together these observations reveal an essential *in vivo* survival function of parkin in close crosstalk with the Ret signaling cascade, converging on mitochondrial integrity control to properly maintain substantia nigra dopaminergic neurons and their innervation in the striatum. This also puts the Ret receptor in an important position in the protein network altered in PD patients.

The identification of mitochondria, the power plants for energy production in all cells including cancer cells, as a common target of parkin and Ret signaling suggests that this crosstalk might not only be important in dopaminergic neurons, but perhaps also in cancer cells. While *increasing* parkin and Ret downstream signaling might be beneficial for PD and Hirschsprung disease patients and cancer patients with a parkin loss of function mutation, *decreasing* Ret signaling should be the strategy for cancer patients with enhanced Ret signaling. Perhaps parkin loss prevents the Ret MEN2 phenotype in mice and man. Further experiments are needed to shed more light on the molecular mechanism of parkin and Ret crosstalk also in the context of cancer.
